# Hedgehog Pathway Is a Regulator of Stemness in HER2-Positive Trastuzumab-Resistant Breast Cancer

**DOI:** 10.3390/ijms252212102

**Published:** 2024-11-11

**Authors:** Idris Er, Asiye Busra Boz Er

**Affiliations:** 1 Department of Medical Biology, Faculty of Medicine, Karadeniz Technical University, Trabzon 61080, Turkey; idriser@ktu.edu.tr; 2 Department of Medical Biology, Faculty of Medicine, Recep Tayyip Erdogan University, Rize 53100, Turkey

**Keywords:** breast cancer, hedgehog, stemness

## Abstract

HER2 overexpression occurs in 20–30% of breast cancers and is associated with poor prognosis. Trastuzumab is a standard treatment for HER2-positive breast cancer; however, resistance develops in approximately 50% of patients within a year. The Hedgehog (Hh) signalling pathway, known for its role in maintaining stemness in various cancers, may contribute to trastuzumab resistance in HER2-positive breast cancer. This study aimed to investigate the role of Hedgehog signalling in maintaining stemness and contributing to trastuzumab resistance in HER2-positive breast cancer cell lines. Trastuzumab-resistant HER2-positive breast cancer cell lines, SKBR3 and HCC1954, were developed through continuous trastuzumab exposure. Cells were treated with GANT61 (Hh inhibitor, IC_50_:10 µM) or SAG21K (Hh activator, IC_50_:100 nM) for 24 h to evaluate the Hedgehog signalling response. Stemness marker expression (*Nanog*, *Sox2*, *Bmi1*, *Oct4*) was measured using qRT-PCR. The combination index (CI) of GANT61 with trastuzumab was calculated using CompuSyn software (version 1.0) to identify synergistic doses (CI < 1). The synergistic concentrations’ impact on stemness markers was assessed. Data were analysed using two-way ANOVA and Tukey’s post hoc test (*p* < 0.05). Trastuzumab-resistant cells exhibited increased Hedgehog signalling activity. Treatment with GANT61 significantly downregulated stemness marker expression, while SAG21K treatment led to their upregulation in both SKBR3-R and HCC1954-R cells. The combination of GANT61 and trastuzumab demonstrated a synergistic effect, markedly reducing the expression of stemness markers. These findings indicate that Hedgehog signalling plays a pivotal role in maintaining stemness in trastuzumab-resistant cells, and that the inhibition of this pathway may prevent tumour progression. Hedgehog signalling is crucial in regulating stemness in trastuzumab-resistant HER2-positive breast cancer. Targeting this pathway could overcome resistance and enhance trastuzumab efficacy. Further studies should explore the clinical potential of Hedgehog inhibitors in combination therapies.

## 1. Introduction

HER2 (Human Epidermal Growth Factor Receptor 2) is overexpressed in 20–30% of breast tumours and is associated with poor prognosis [1]. Trastuzumab is an FDA-approved treatment for HER2-positive breast cancer that reduces HER2 overactivity through various mechanisms, including preventing HER2 dimerization, downregulating the HER2 receptor via endocytic destruction, and inhibiting HER2 cleavage by metalloproteases [2]. Nevertheless, resistance is a major issue, with around 50% of HER2-positive breast cancer patients developing resistance to trastuzumab within a year of treatment [3].

Trastuzumab resistance in HER2-positive cancers arises from several mechanisms. One key factor is alterations in the HER2 receptor, such as truncated forms like p95HER2, which lack the extracellular domain targeted by trastuzumab, rendering it ineffective [4]. Increased HER2 expression or amplification can also saturate the drug’s capacity to block all receptors. Another factor is the activation of alternative signalling pathways, like the PI3K/AKT/mTOR pathway that is often driven by PIK3CA mutations, which bypass HER2 inhibition while HER family receptors such as HER3 or EGFR can continue oncogenic signalling [5]. The dysregulation of downstream signalling, including PTEN loss, further enhances resistance by hyperactivating these pathways. Epigenetic changes and immune evasion also play roles in altering HER2 signalling and diminishing trastuzumab’s efficacy by reducing antibody-dependent cell-mediated cytotoxicity (ADCC) [6]. Additionally, the upregulation of stemness pathways (e.g., Notch, Hedgehog, Wnt) and changes in cell surface integrins enable cancer cells to bypass HER2 reliance [7]. Lastly, trastuzumab internalization and degradation within lysosomes limit its availability over time [8]. Together, these mechanisms create a complex resistance landscape, demanding new therapeutic approaches [9]. Enhanced HER2–HER3 dimerization also promotes cell survival and proliferation. Additionally, the tumour microenvironment, including hypoxia and the presence of cancer stem cells (CSCs), contributes to drug resistance.

“Stemness” in cancer refers to the ability of cancer cells to exhibit stem cell-like characteristics, such as self-renewal, differentiation potential, and resistance to conventional therapies [10]. This is critical for tumour progression, metastasis, recurrence, and drug resistance [11]. Previous studies have shown that CSCs contribute to tumour initiation and maintenance and are often involved in therapeutic resistance.

Hedgehog signalling plays a crucial role in sustaining a subset of tumour cells with stem cell-like properties [12]. These CSCs are believed to maintain a self-renewing reservoir while differentiating into transient amplifying cells, thereby creating a heterogeneous tumour environment [13,14]. The Hedgehog signalling pathway is thought to drive the CSC phenotype by improperly regulating stemness-related genes. For instance, Nanog, a key transcription factor involved in both the self-renewal of embryonic stem cells and the reprogramming of somatic cells to a pluripotent state, is directly targeted by Hh signalling [15]. Additionally, Hh signalling upholds a stemness signature in various cancers by promoting the expression of other stemness-regulating genes, such as Oct4, Sox2, and Bmi1 [16,17,18].

Aberrant activation of the Hedgehog pathway has been observed in various cancers, including breast cancer, and is associated with increased tumour aggressiveness and resistance to therapy [17]. However, its effect on stemness in HER2-positive, trastuzumab-resistant breast cancer remains unclear. In this aspect, our study investigated the relationship between Hedgehog signalling and stemness gene expression, identifying the Hedgehog pathway as a key signalling mechanism responsible for stemness in HER2-resistant breast cancer.

## 2. Results

### 2.1. The Hedgehog Pathway Is Responsive in Trastuzumab-Resistant Breast Cancer Cell Lines

**Figure 1:**
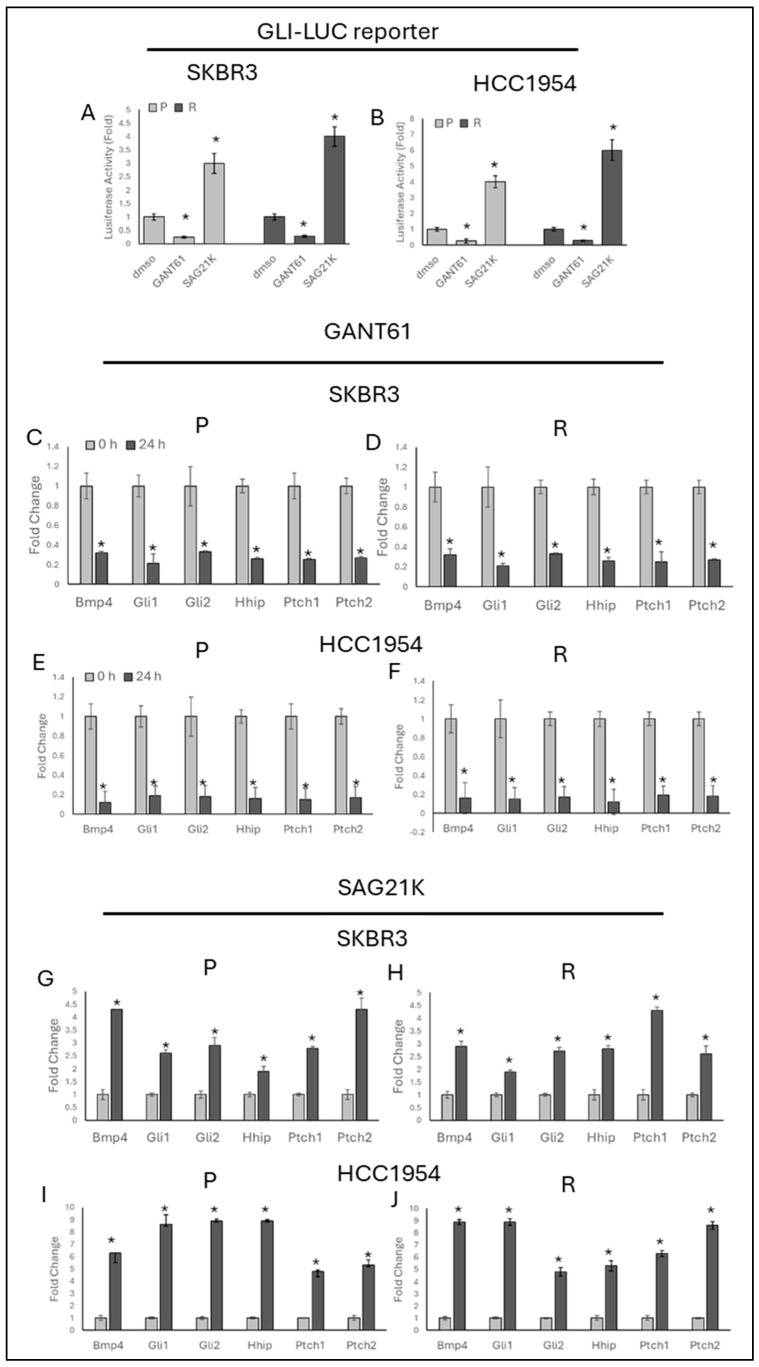
GANT61 decreases Hedgehog signalling while SAG21K increases it in resistant SKBR3 cells. Luciferase activity of the GLI-LUC reporter decreased in the presence of GANT61 and increased in the presence of SAG21K in both parental and resistant cells. The data are presented as follows: (**A**) SKBR3 and (**B**) HCC1954 cells showing GLI-LUC activity; (**C**) GANT61 treatment decreasing Hedgehog-responsive genes in SKBR3-P, (**D**) SKBR3-R, (**E**) HCC1954-P, and (**F**) HCC1954-R cells; (**G**) SAG21K treatment increasing Hedgehog-responsive genes in SKBR3-P, (**H**) SKBR3-R, (**I**) HCC1954-P, and (**J**) HCC1954-R cells. Student’s *t*-test was used for statistical analysis. * *p* ≤ 0.05, *n* = 3 ± SD.

To observe Hedgehog activity in the trastuzumab-resistant SKBR3 and HCC1954 cell lines, GANT61 was used to inhibit while SAG21K was used to activate the Hedgehog pathway, with DMSO serving as the control. In both the SKBR3-R and HCC1954-R (Figure 1) cells, Hedgehog-responsive gene expression (*Bmp4*, *Gli1*, *Gli2*, *Hhip*, *Ptch1*, *Ptch2*) decreased with GANT61 treatment and increased with SAG21K treatment after 24 h, as shown in the luciferase reporter assay and real-time PCR.

### 2.2. Hedgehog Inhibition and Activation Regulate Stemness Markers in Trastuzumab-Resistant Breast Cancer Cell Lines

**Figure 2:**
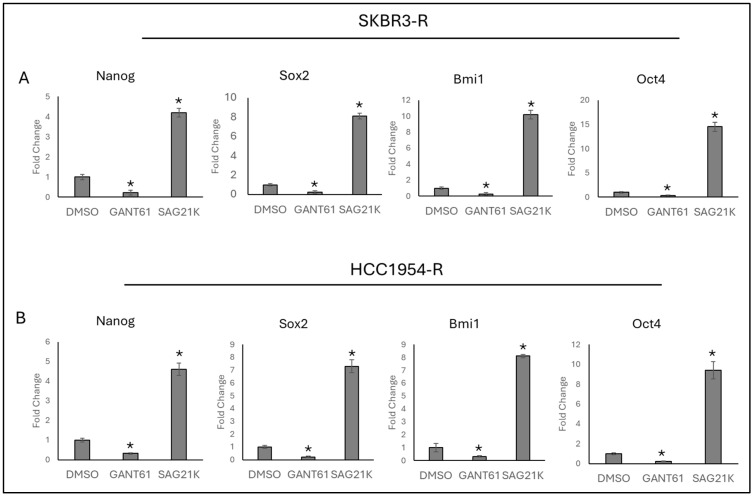
Hedgehog inhibition and activation regulate stemness markers in trastuzumab-resistant breast cancer cell lines SKBR3-R and HCC1954-R. (**A**) Treatment with GANT61 reduces the expression of stemness marker genes, while SAG21K induces their expression in (**A**) SKBR3-R and (**B**) HCC1954-R cell lines. R: resistant, (*n* = 3 ± SD) Statistical analysis was carried out using ANOVA variation test and Tukey’s post hoc test to show significance. Differences are considered significant * *p* ≤ 0.05.

To observe the effect of Hedgehog signalling on stemness markers in trastuzumab-resistant cell lines, the SKBR3-R and HCC1954-R cells were treated with GANT61 and SAG21K. The expression of stemness markers *Nanog*, *Sox2*, *Bmi1*, and *Oct4* was analysed. It was observed that the inhibition of Hedgehog signalling decreased the expression of *Nanog*, *Sox2*, *Bmi1*, and *Oct4*, while the activation of Hedgehog signalling increased their expression in both the SKBR3-R (Figure 2A) and HCC1954-R (Figure 2B) cells.

### 2.3. Trastuzumab+GANT61 Combination Has Synergistic Effect on Trastuzumab-Resistant HER2 + Breast Cancer Cell Lines

**Figure 3:**
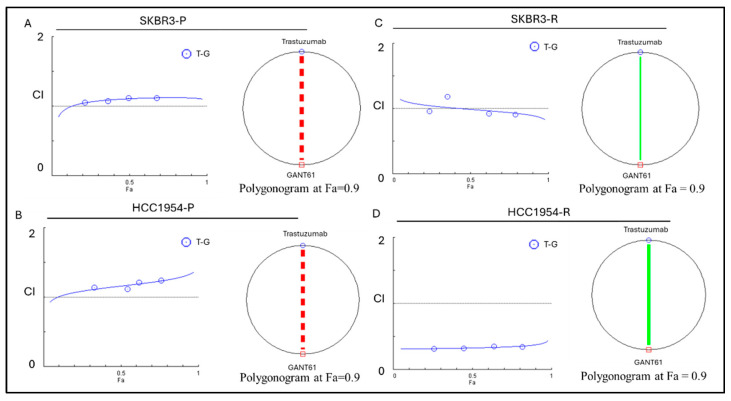
Trastuzumab and GANT61 combination therapy results for combination index plot and polygonogram for SKBR3-P, HCC1954-P, SKBR3-R and HCC1954-R. The red line in (**A**) SKBR3-R and (**B**) HCC1954-P polygonogram indicates strong antagonist, The green line with specific thickness in (**C**) SKBR3-R polygonogram indicates a slightly synergism effect; (**D**) HCC1954-P polygonogram indicates a synergistic effect. Fa: Fraction affected; Fa = 0.9 means 90% cell death; R: resistant; CI: combination index; CI > 1.1 represents antagonism; CI < 0.9 represents synergism; CI 0.9–1.1 represents an additive effect.

To assess the combined effect of trastuzumab+GANT61 on cells, the results were obtained using the following five concentration points at a 1:1 ratio for each drug: 0.25-fold, 0.5-fold, 1-fold, 2-fold, and 4-fold of the IC_50_ values for each individual drug. The IC_50_ values used in the experiments are listed in materials and methods. The combination effect was evaluated by calculating the combination index (CI) using the median effect principle established by Chou and Talalay [19]. The line type in the polygonogram represents the quantification of synergism (Figure 3). The results indicate that the trastuzumab+GANT61 drug combination is a strong antagonist in the SKBR3-P and HCC1954-P cell lines, very strongly synergistic in the SKBR3-R cell line, and synergistic in the HCC1954-R cell line (Table 1).

### 2.4. Trastuzumab+GANT61 Combination Synergistically Decrease Stemness Markers in Trastuzumab-Resistant HER2 + Breast Cancer Cell Lines

In the combination therapy, the synergistic doses of trastuzumab and GANT61 were determined to be 0.2 and 0.4 for SKBR3-R cells and 0.6 and 0.5 for HCC1954-R cells, respectively. To assess the effect of these synergistic doses on stemness marker expression, high doses of trastuzumab and GANT61 were used both as monotherapies and in combination and compared with the synergistic doses of trastuzumab and GANT61 in similar settings. For high-dose treatment, 1 µM of both trastuzumab and GANT61 was applied to the SKBR3-R and HCC1954-R cells, while the synergistic doses were 0.2 and 0.4 for SKBR3-R and 0.6 and 0.5 for HCC1954-R.

Results showed that 1 µM GANT61 monotherapy, the combination of 1 µM GANT61 with trastuzumab, and the synergistic dose combination of GANT61 and trastuzumab significantly reduced the expression of *Nanog*, *Sox2*, *Bmi1*, and *Oct4* in the SKBR3-R and HCC1954-R cells. In the SKBR3-R cells, the high-dose trastuzumab and GANT61 combination led to a decrease of approximately 27-fold for *Nanog*, 15-fold for *Sox2*, 12-fold for *Bmi1*, and 12-fold for *Oct4*, compared to the DMSO control. Notably, the synergistic doses of trastuzumab and GANT61 reduced *Nanog*, *Bmi1*, and *Oct4* by 5-fold, and *Sox2* by 10-fold, compared to the high-dose combination in the SKBR3-R cells.

**Figure 4:**
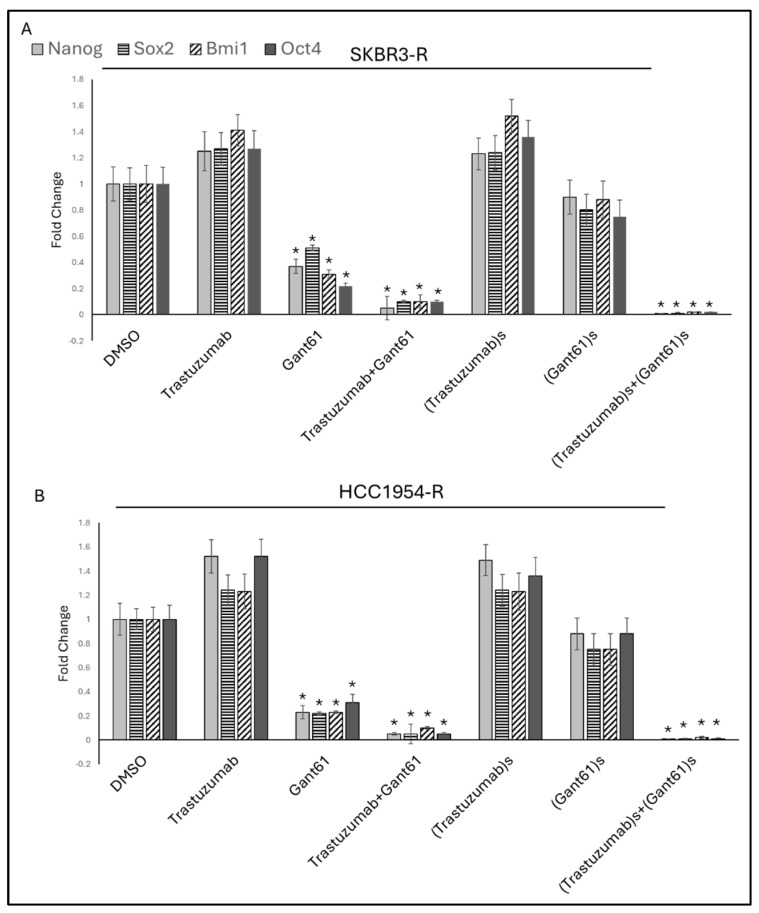
Trastuzumab+GANT61 combination synergistically decrease stemness markers *Nanog*, *Sox2*, *Bmi1*, *Oct4*, 1 µM GANT61 monotherapy, 1 µM GANT61+trastuzumab combination, and synergistic dose of GANT61+trastuzumab combination significantly decreased *Nanog*, *Sox2*, *Bmi1*, *Oct4* (**A**) in SKBR3-R, (**B**) in HCC1954-R cells. s(Trastuzumab): Synergistic dose of trastuzumab in combination with GANT61, s(GANT61): Synergistic dose of GANT61 in combination with trastuzumab. R: resistant; (*n* = 3 ± SD) Statistical analysis was carried out using ANOVA variation test and Tukey’s post hoc test to show significance. Differences are considered significant * *p* ≤ 0.05.

Similarly, in the HCC1954-R cells, the high-dose combination of trastuzumab and GANT61 reduced *Nanog*, *Sox2*, and *Oct4* by approximately 30-fold, and *Bmi1* by around 15-fold, compared to DMSO. Interestingly, the synergistic doses of trastuzumab and GANT61 reduced *Nanog*, *Bmi1*, *Oct4*, and *Sox2* by 5-fold when compared to the high-dose combination in the SKBR3-R cells (Figure 4).

## 3. Discussion

The critical role of the Hedgehog (Hh) signalling pathway in maintaining stemness in trastuzumab-resistant HER2-positive breast cancer cell lines was highlighted by this study, suggesting that targeting this pathway could be a promising strategy to overcome resistance and enhance the efficacy of existing therapies. It was demonstrated that trastuzumab-resistant cells exhibited upregulated Hedgehog signalling, which correlated with the increased expression of stemness markers such as *Nanog*, *Sox2*, *Bmi1*, and *Oct4*.

The inhibition of Hedgehog signalling with GANT61 was found to significantly reduce the expression of these stemness markers, whereas activation with SAG21K led to their upregulation in both the SKBR3-R and HCC1954-R cell lines. This finding underscores the importance of Hedgehog signalling in sustaining a stem cell-like phenotype in resistant breast cancer cells. These results align with previous studies that suggest aberrant Hedgehog signalling contributes to tumour aggressiveness and resistance to therapy by maintaining cancer stem cell properties [11].

Furthermore, a synergistic effect was demonstrated by the combination of GANT61 and trastuzumab in downregulating stemness markers, significantly more so than high-dose monotherapies. The expressions of *Nanog*, *Sox2*, *Bmi1*, and *Oct4* were reduced to a greater extent by the synergistic doses of GANT61 and trastuzumab than by the high-dose combination, suggesting that lower doses of the combined therapy could effectively diminish the stemness characteristics in resistant cells. These findings suggest that Hedgehog inhibitors, when combined with trastuzumab, could potentiate anti-cancer effects and help overcome drug resistance.

The differential impact of the combination therapy on stemness marker expression in SKBR3-R and HCC1954-R cells suggests that the level of Hedgehog pathway activation and the underlying molecular mechanisms of resistance may vary between different HER2-positive breast cancer cell lines. This variability may be attributed to differences in the tumour microenvironment, genetic mutations, or alternative pathway activation, such as the PI3K/AKT or MAPK pathways. It is suggested that future studies explore these aspects to better understand the context-dependent roles of Hedgehog signalling in resistance mechanisms.

Significant clinical implications are indicated by these findings. Given that trastuzumab resistance is a major challenge in treating HER2-positive breast cancer, the Hedgehog pathway presents a potential target to enhance trastuzumab efficacy and overcome resistance. Hedgehog inhibitors like GANT61 could be developed as adjuvant therapies to target CSCs and reduce tumour recurrence, especially in patients exhibiting poor responses to trastuzumab alone. Additionally, it is suggested by our study that combining Hedgehog inhibitors with trastuzumab may require lower doses, potentially minimizing adverse effects while maximizing therapeutic benefits.

However, certain limitations to this study should be acknowledged. First, the findings are based on in vitro models, which may not fully recapitulate the complexities of tumour behaviour in vivo. Therefore, further preclinical and clinical studies are necessary to confirm these results and determine the safety and efficacy of combining Hedgehog inhibitors with trastuzumab in patients. Additionally, the study focuses on specific stemness markers; it would be beneficial for future research to explore additional markers and pathways involved in trastuzumab resistance to provide a more comprehensive understanding of the mechanisms at play.

Clinical trials specifically targeting GANT61, a GLI inhibitor for Hedgehog signalling, are limited, and much of the research remains preclinical, focusing on various cancer models. GANT61 has shown promising results in laboratory studies, particularly for cancers with aberrant Hedgehog pathway activation, such as glioblastoma [20], mantle cell lymphoma [21], and melanoma [22].

For instance, GANT61 was used to inhibit GLI1 in combination with a Wnt pathway inhibitor, showing enhanced apoptosis and drug sensitivity in mantle cell lymphoma models [21]. Similarly, in glioblastoma, GANT61 significantly reduced the cell viability, proliferation, and migration of tumour spheroids when paired with conditioned media from reactive astrocytes, indicating the drug’s potential for tackling aggressive tumours with high GLI1 expression [23]. These studies indicate GANT61′s potential as part of combination therapy strategies, especially when paired with other pathway inhibitors.

In conclusion, evidence has been provided by this study that the Hedgehog pathway is a key regulator of stemness in trastuzumab-resistant HER2-positive breast cancer. Targeting this pathway in combination with trastuzumab may offer a promising strategy to overcome resistance and improve patient outcomes. It is recommended that future research focus on further elucidating the mechanisms by which Hedgehog signalling contributes to drug resistance and exploring the therapeutic potential of Hedgehog inhibitors in combination with current standard-of-care treatments.

## 4. Materials and Methods

### 4.1. Cell Culture

HCC1954 (ATCC Cat#CRL2338) and SKBR3 (ATCC Cat#HTB30) are HER2-positive breast cancer cell lines purchased from the American Type Culture Collection. The cell lines were grown in DMEM media supplemented with 10% FBS (Merck, Rahway, NJ, USA), 1% sodium pyruvate (Merck, Rahway, NJ, USA), and 2 mM L-glutamine (Merck, Rahway, NJ, USA). HCC1954 and SKBR3 trastuzumab-resistant cell lines were generated by exposing the cells to increased doses (0.1–10 μM) of trastuzumab for 3 months. The protocol and confirmation can be found in our previous paper [24]. SAG21K (5282-Tocris-Bristol-UK) is a Hedgehog activator [25] that targets smoothened protein and was used as a positive control, while GANT61 (S8075-Selleckchem-Houston-TX-USA) is a GLI inhibitor [12,26,27,28] that decreases Hedgehog signalling and was used as a negative control. Approximately 1 µM each of GANT61 and SAG21K were used, and the cells were incubated for 24 h before RNA isolation.

### 4.2. Luciferase Reporter Assay

GLI, as a Hedgehog responsive reporter plasmid, was used to show the activation of Hedgehog. GANT61 and SAG21K were used as positive and negative controls, respectively. The pCMV-β-Gal plasmid was generously provided by Talat Nasim from the University of Bradford, UK, and the pMuLE_ENTR_12GLI-FLuc_R4-R3 plasmid was obtained from Manfred Ogris [29] (Addgene plasmid # 113712; http://n2t.net/addgene:113712-access date 1 July 2024).

Cells were lysed 24 h after transfection with a reporter lysis buffer (Promega, Cat. No. E4030) and luciferase activity was measured by a luminometer (Fluoroskan ascent FL-Thermo Scientific-Waltham, WA, USA) immediately after dispensing a luciferase assay system substrate luciferin by the luminometer.

The luciferase activity was normalized for transfection efficiency by β-galactosidase activity and quantified by absorption at 405 nm after incubation with ONPG (4 mg/mL) (Merck, Rahway, NJ, USA) + β-mercaptoethanol (Merck, Rahway, NJ, USA) + Z buffer, and the reaction was terminated with 1 M Na_2_CO_3_ (Merck, Rahway, NJ, USA) buffer.

The reporter assay data were divided by the β-galactosidase assay results to normalize the transfection efficiency.

### 4.3. Combination Therapy

**Figure 5:**
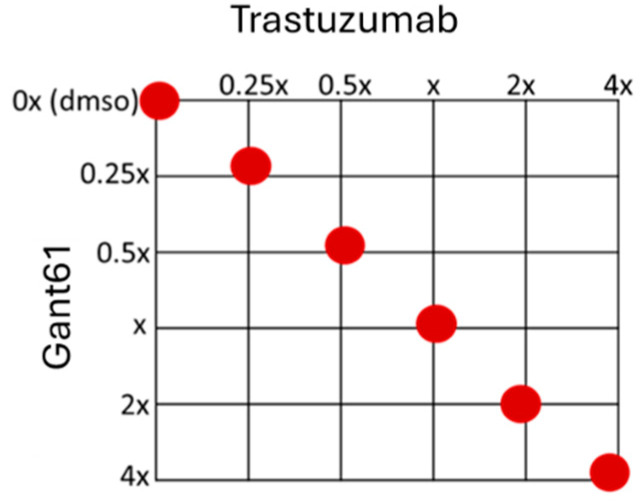
Checkerboard of the trastuzumab+GANT61 combination therapy experiment. Red dots show the concentrations tested as multiples of IC_50_.

The SKBR3-R and HCC1954-R cells were treated with GANT61 combined with trastuzumab in a 1:1 ratio of concentrations such as 0.25-fold, 0,5-fold, 1-fold, 2-fold, and 4-fold IC_50_ of the individual drugs (Figure 5), and the effect of the combination was analysed by an MTT assay. The CompuSyn software (Version 1.0) was used to calculate the combination index (CI), which indicated the interaction between the drugs. CI > 1.1 represents antagonism; CI < 0.9 represents synergism; and CI 0.9–1.1 represents an additive effect [30].

### 4.4. Quantitative Real-Time PCR

RNA was isolated using the Qiagen RNeasy kit according to the manufacturer’s instructions. cDNA synthesis was carried out with the Biorad (Hercules-CA-USA) iScript Reverse Transcription Supermix for RT-qPCR. The PCR was performed using the iTaq Universal SYBR Green One-Step Kit (Biorad, Hercules-CA-USA), and the results were measured with the Applied Biosystems ABI 7500 Real-Time PCR Instrument (Applied Biosystems, Waltham, WA, USA) and 7500 software v1. The amplification conditions were 95 °C for 10 s and 60 °C for 1 min, for 40 cycles, with melting conditions of denaturation at 95 °C for 15 s, annealing at 60 °C for 1 min, and elongation at 95 °C for 15 s. Real-time primers (*Oct4*, *Sox2*, *Nanog*, *Bmi1*, *GAPDH*, *Bmp4*, *Gli1*, *Gli2*, *Hhip*, *Ptch1*, *Ptch2*) were obtained from Sigma KiqStart. Each PCR reaction was conducted in triplicate, and the experiments were repeated three times with different samples. To analyse the RT-qPCR data, the Ct values of cDNA were normalized to the housekeeping gene GAPDH. The data were processed using the formula ΔCt = Ct (target gene) − Ct (housekeeping gene). Fold changes were calculated using the 2^–ΔΔCt^ method, where 2^–ΔCt^ (sample)/2^–ΔCt^ (control) was used to determine the fold changes [31,32].

### 4.5. Statistical Analysis

Statistical analysis was performed using a two-tailed Student’s *t*-test, two-way ANOVA, and Tukey’s post hoc test to assess significance, with a *p* * ≤ 0.05 value deemed significant. Error bars represent ± SD from three independent experiments, each conducted in triplicate. Details of the analytical methods and *p*-values are indicated in the figure legends.

## 5. Conclusions

Our study highlights the significant role of Hedgehog signalling in regulating stemness in trastuzumab-resistant HER2-positive breast cancer cell lines. By demonstrating that the modulation of this pathway affects the expression of key stemness markers, we propose that targeting Hedgehog signalling could serve as a novel approach to mitigate resistance and improve the efficacy of trastuzumab in HER2-positive breast cancer treatment. Further research is warranted to explore the clinical potential of Hedgehog inhibitors in combination with trastuzumab and to elucidate the detailed mechanisms underlying Hedgehog-mediated stemness in resistant breast cancer cells.

## Figures and Tables

**Table 1:** Trastuzumab and GANT61 combination therapy results in CI value. R: resistant; CI: combination index; CI > 1.1 represents antagonism; CI < 0.9 represents synergism; CI 0.9–1.1 represents an additive effect.

Cell Lines	Drug Combo CI Value	Description of Effect
SKBR3-P	2.77	Strong antagonism
SKBR3-R	0.87	Slight synergism
HCC1954-P	2.43	Strong antagonism
HCC1954-R	0.35	Synergism

## Data Availability

The datasets and materials used and/or analyzed during the current study are available from the corresponding author upon reasonable request.

## References

[1] Burnett J.P., Korkaya H., Ouzounova M.D., Jiang H., Conley S.J., Newman B.W., Sun L., Connarn J.N., Chen C.S., Zhang N. (2015). Trastuzumab resistance induces EMT to transform HER2 (+) PTEN (−) to a triple negative breast cancer that requires unique treatment options. Sci. Rep..

[2] Bose R., Ma C.X. (2021). Breast Cancer, HER2 Mutations, and Overcoming Drug Resistance. N. Engl. J. Med..

[3] Luque-Bolivar A., Perez-Mora E., Villegas V.E., Rondon-Lagos M. (2020). Resistance and Overcoming Resistance in Breast Cancer. Breast Cancer Targets Ther..

[4] Zagozdzon R., Gallagher W.M., Crown J. (2011). Truncated HER2: Implications for HER2-targeted therapeutics. Drug Discov. Today.

[5] Prasad D., Baldelli E., Blais E.M., Davis J., El Gazzah E., Mueller C., Gomeiz A., Ibrahim A., Newrekar A.V., Corgiat B.A. (2024). Functional activation of the AKT-mTOR signalling axis in a real-world metastatic breast cancer cohort. Br. J. Cancer.

[6] Khan S.U., Fatima K., Aisha S., Malik F. (2024). Unveiling the mechanisms and challenges of cancer drug resistance. Cell Commun. Signal.

[7] Takebe N., Miele L., Harris P.J., Jeong W., Bando H., Kahn M., Yang S.X., Ivy S.P. (2015). Targeting Notch, Hedgehog, and Wnt pathways in cancer stem cells: Clinical update. Nat. Rev. Clin. Oncol..

[8] Maass K.F., Kulkarni C., Betts A.M., Wittrup K.D. (2016). Determination of Cellular Processing Rates for a Trastuzumab-Maytansinoid Antibody-Drug Conjugate (ADC) Highlights Key Parameters for ADC Design. AAPS J..

[9] Hayes D.F. (2019). HER2 and Breast Cancer—A Phenomenal Success Story. N. Engl. J. Med..

[10] Aponte P.M., Caicedo A. (2017). Stemness in Cancer: Stem Cells, Cancer Stem Cells, and Their Microenvironment. Stem Cells Int..

[11] Chang W.H., Lai A.G. (2019). Aberrations in Notch-Hedgehog signalling reveal cancer stem cells harbouring conserved oncogenic properties associated with hypoxia and immunoevasion. Br. J. Cancer.

[12] Harada K., Ohashi R., Naito K., Kanki K. (2020). Hedgehog Signal Inhibitor GANT61 Inhibits the Malignant Behavior of Undifferentiated Hepatocellular Carcinoma Cells by Targeting Non-Canonical GLI Signaling. Int. J. Mol. Sci..

[13] Chen K., Huang Y.H., Chen J.L. (2013). Understanding and targeting cancer stem cells: Therapeutic implications and challenges. Acta Pharmacol. Sin..

[14] Medema J.P. (2013). Cancer stem cells: The challenges ahead. Nat. Cell Biol..

[15] Po A., Ferretti E., Miele E., De Smaele E., Paganelli A., Canettieri G., Coni S., Di Marcotullio L., Biffoni M., Massimi L. (2010). Hedgehog controls neural stem cells through p53-independent regulation of Nanog. EMBO J..

[16] Clement V., Sanchez P., de Tribolet N., Radovanovic I., Altaba A.R.I. (2007). HEDGEHOG-GLI1 signaling regulates human glioma growth, cancer stem cell self-renewal, and tumorigenicity. Curr. Biol..

[17] Justilien V., Walsh M.P., Ali S.A., Thompson E.A., Murray N.R., Fields A.P. (2014). The PRKCI and SOX2 oncogenes are coamplified and cooperate to activate Hedgehog signaling in lung squamous cell carcinoma. Cancer Cell.

[18] Cochrane C.R., Szczepny A., Watkins D.N., Cain J.E. (2015). Hedgehog Signaling in the Maintenance of Cancer Stem Cells. Cancers.

[19] Chou T.C. (2010). Drug combination studies and their synergy quantification using the Chou-Talalay method. Cancer Res..

[20] Mubeena Mariyath P.M., Farheen S., Sharma R.M., Shahi M.H. (2024). Differential regulation of Shh-Gli1 cell signalling pathway on homeodomain transcription factors Nkx2.2 and Pax6 during the medulloblastoma genesis. Mol. Biol. Rep..

[21] Han Y., Li C., Liu S., Gao J., He Y., Xiao H., Chen Q., Zheng Y., Chen H., Zhu X. (2024). Combined targeting of Hedgehog/GLI1 and Wnt/beta-catenin pathways in mantle cell lymphoma. Hematol. Oncol..

[22] Vlckova K., Reda J., Ondrusova L., Krayem M., Ghanem G., Vachtenheim J. (2016). GLI inhibitor GANT61 kills melanoma cells and acts in synergy with obatoclax. Int. J. Oncol..

[23] Ribeiro J.H., Villarinho N.J., Fernandes P.V., Spohr T., Lopes G.P.F. (2024). Conditioned Medium from Reactive Astrocytes Inhibits Proliferation, Resistance, and Migration of p53-Mutant Glioblastoma Spheroid Through GLI-1 Downregulation. J. Cell Biochem..

[24] Er A.B.B. (2024). Integrin β3 Reprogramming Stemness in HER2-Positive Breast Cancer Cell Lines. Biology.

[25] He L., Sato J.E., Sundar P., Azimi T., Beachy P.A., Bekale L.A., Pepper J.P. (2024). Localized application of SAG21k-loaded fibrin hydrogels for targeted modulation of the hedgehog pathway in facial nerve injury. Int. J. Biol. Macromol..

[26] de Araújo T.B.S., Rocha L.d.O.S.d., Vidal M.T.A., Coelho P.L.C., dos Reis M.G., Souza B.S.d.F., Soares M.B.P., Pereira T.A., Della Coletta R., Bezerra D.P. (2020). GANT61 Reduces Hedgehog Molecule (GLI1) Expression and Promotes Apoptosis in Metastatic Oral Squamous Cell Carcinoma Cells. Int. J. Mol. Sci..

[27] Er A.B.B., Er I. (2024). Targeting ITGβ3 to Overcome Trastuzumab Resistance through Epithelial–Mesenchymal Transition Regulation in HER2-Positive Breast Cancer. Int. J. Mol. Sci..

[28] Er A.B.B., Er I. (2024). PAI1 Regulates Cell Morphology and Migration Markers in Trastuzumab-Resistant HER2-Positive Breast Cancer Cells. Life.

[29] Maier J., Elmenofi S., Taschauer A., Anton M., Sami H., Ogris M. (2019). Luminescent and fluorescent triple reporter plasmid constructs for Wnt, Hedgehog and Notch pathway. PLoS ONE.

[30] Chou T.-C. (2018). The combination index (CI < 1) as the definition of synergism and of synergy claims. Synergy.

[31] Schmittgen T.D., Livak K.J. (2008). Analyzing real-time PCR data by the comparative C (T) method. Nat. Protoc..

[32] Delgado-Enciso I., Paz-Garcia J., Rodriguez-Hernandez A., Madrigal-Perez V.M., Cabrera-Licona A., Garcia-Rivera A., Soriano-Hernandez A.D., Cortes-Bazan J.L., Galvan-Salazar H.R., Valtierra-Alvarez J. (2018). A promising novel formulation for articular cartilage regeneration: Preclinical evaluation of a treatment that produces SOX9 overexpression in human synovial fluid cells. Mol. Med. Rep..

